# Heterotopic Pregnancy Secondary to *in vitro* Fertilization-Embryo Transfer: Risk Factors and Pregnancy Outcomes

**DOI:** 10.3389/fmed.2022.864560

**Published:** 2022-06-22

**Authors:** Shaomi Zhu, Yiyue Fan, Ling Lan, Tianqing Deng, Qinxiu Zhang

**Affiliations:** School of Medical and Life Sciences/Reproductive & Women-Children Hospital, Chengdu University of Traditional Chinese Medicine, Chengdu, China

**Keywords:** ectopic pregnancy, heterotopic pregnancy, *in vitro* fertilization, pregnancy outcome, intrauterine pregnancy

## Abstract

**Background:**

With the widespread application of assisted reproduction technology (ART) and increased pelvic inflammatory disease, the incidence of heterotopic pregnancy (HP) has risen. However, the risk factors and treatment of HP remain indefinite.

**Objectives:**

To explore risk factors affecting the incidence of HP secondary to *in vitro* fertilization-embryo transfer (IVF-ET) and pregnancy outcomes after surgical treatment of HP.

**Methods:**

29 patients with HP and 116 with an intrauterine-only pregnancy (IUP) after IVF-ET during the same period were included retrospectively from January 2015 to September 2020.

**Results:**

Patients with HP had a higher proportion of previous ectopic pregnancies, multiple abortion history (≧2 times) and tubal indication for IVF than IUP. Besides, they had a greater possibility to end in spontaneous abortion (31.03 vs.13.79%, *P* = 0.028) and preterm delivery (25.00 vs. 7.00%, *P* = 0.035), less possibility to result in a live birth (58.62 vs. 78.45%, *P* = 0.028). History of multiple abortions (≥2 times) [odds ratio (OR) 3.031, 95% confidence intervals (CI) 1.087–8.453; *P* = 0.034], tubal infertility (OR 3.844, 95% CI 1.268–11.656; *P* = 0.017), previous ectopic pregnancies (OR 2.303, 95% CI 0.625–8.490; *P* = 0.021) and number of embryo transfer (OR 0.300, 95% CI 0.092–0.983; *P* = 0.037) resulted in an elevated proportion of HP in IVF treatment. Shorter operative duration, smaller size of the ectopic mass and location in the ampulla of the fallopian tube were associated with higher chance of survival in the coexistent intrauterine pregnancy after surgical treatment.

**Conclusions:**

Previous history of ectopic pregnancy, multiple abortions, tubal infertility and multiple-embryo transfer may be considered as meaningful risk factors of subsequent HP following IVF-ET. In patients with HP treated by surgery, shorter operative duration, smaller size of the ectopic mass and location in the ampulla of the fallopian tube means better reproductive prognosis.

## Introduction

HP, defined as simultaneous occurrence of ectopic and intrauterine pregnancy, is a rare disease in the general population. Previously, the incidence rate of HP was estimated from previous study between 1/7,963 and 1/30,000 in spontaneous pregnancies (1). However, the incidence has risen, ranging from 1.5 in 1,000 to 1 in 100 (2), with the widespread application of assisted reproduction technology (ART) and increased pelvic inflammatory disease. The early diagnosis and treatment of HP remain one of the biggest challenges in the field of gynecology. A coexisting intrauterine gestation makes the ectopic pregnancy in HP more difficult to diagnose. However, delay in diagnosis may increase the probability of tubal rupture, resulting in hemorrhagic shock and emergency blood transfusion (3). Therefore, enhanced follow-up care should be performed in patients considered to be at high risk for HP. However, very little definite data exists regarding risk factors for HP after IVF because of the rareness of the disease. Several probable explanations for HP are suggested in recent years, such as transfer of multiple embryos, embryo transferring directly into the oviduct and transplantation in large volume of medium (4).

The ideal treatment of HP is to remove the ectopic pregnancy with least harm to the intrauterine gestational sac. It is well known that surgical treatment with HP is laparoscopy or laparotomy. In recent years, more and more patients with HP prefer to laparoscopy because of the minimally invasive approach (5). Therefore, factors affecting prognosis after surgical therapy of HP are attention-attracting topic. However, clinical data addressing this issue are limited. The surgical characteristics associated with survival of the intrauterine pregnancy after surgical treatment with HP are not yet known.

The purpose of the present retrospective study was to detect the risk factors of HP for patients pregnant following ART treatment. It also addressed the factors influencing the pregnancy outcome following the surgical treatment of HP.

## Method

### Study Population and Design

From January 2015 to September 2020, 4,274 cycles following IVF-ET at the ART Center in Reproductive &Women-Children Hospital of Chengdu University of Traditional Chinese Medicine resulted in a pregnancy (clinical intrauterine, ectopic and heterotopic). Twenty nine patients were diagnosed with HP and included in this study. Among the 29 patients with HP, 2 had a spontaneous miscarriage of the intrauterine pregnancy, and then were treated with methotrexate for the ectopic pregnancy. In another case, spontaneous resolution of the extrauterine gestation occurred, and the intrauterine pregnancy failed to survive after 2 weeks. One patient with cervical ectopic pregnancy was successfully treated with ultrasound-guided aspiration. The remaining 25 patients with HP received surgical treatment. During the same period, 116 patients with an intrauterine-only pregnancy after embryo transfer were enrolled as controls from the IVF registry system in the ART center.

IUP was defined as one or more gestational sacs located solely inside the uterus and visible by ultrasound. HP was defined as the simultaneous visualization of both intrauterine and extrauterine pregnancy. Diagnosis of HP was confirmed on postsurgical pathology in the present study. In our hospital, patients treated with IVF are required to return for a blood β-human chorionic gonadotrophin (β-hCG) test 14 and 18 days after embryo transfer. The patients who have elevated β-hCG levels will come back for transvaginal ultrasound examination 30 days after embryo transfer. After surgical treatment, patients with HP received intensive treatment with the goal of maintaining the intrauterine pregnancy. Besides, a weekly follow-up ultrasound scan was performed during the first trimester of pregnancy.

Maternal characteristics including body mass index (BMI), age, infertility diagnosis and previous pregnancy history were assessed. ART procedure characteristics: fresh or frozen-thawed cycles, number of embryos transferred, cleavage or blastocyst embryos, endometrial thickness and distance of air bubble to fundus were also assessed. In addition, the surgical characteristics were analyzed. The pregnancy outcomes of patients with HP and IUP were compared in this study. The survival rate of intrauterine pregnancy was defined as the rate of intrauterine gestational sac surviving after 12 weeks of gestation in surgically treated patients with HP. Spontaneous abortion was defined as embryonic/fetal loss before 20-week gestation. Preterm delivery was defined as delivery before 37 completed weeks.

In IVF centers, the follow-up time points including 14, 28 days, 12 weeks after embryo transplantation and perinatal period. The frequency of follow-up will be increased under some abnormal conditions. Patients with poor HCG doubling should be suggested to have shorter ultrasound intervals for earlier detection of ectopic pregnancy. Follow-up is mainly completed by telephone and outpatient clinic. The follow-up must be completed at the time around the time point without delay. Follow-up results were recorded in the IVF register system in time. In the present study, all data was extract from the register system including copies of hospital admission and surgical records in patients with HP at the time of diagnosis and treatment. This information is timely and accurate, minimizing the follow-up bias. The current study was approved by the hospital ethics committee. In our center, patients have permitted the use of their medical records for research by signing a written informed consent before IVF treatment and the program was according to Helsinki Declaration.

### Statistical Analysis

All statistical analysis were performed using the SPSS 19.0 software. A multivariate logistic regression modal was performed to obtain relative risk factors for HP. The *T*- test was used when variables obeyed a normal distribution. Chi-square tests were used to compare data shown as percentage. *P*-values < 0.05 were considered significant.

## Results

### Maternal Characteristics and Pregnancy Outcome

Among the 145 patients included in our study, no significant differences were detected between patients with HP and IUP with respect to BMI, maternal age, infertility diagnosis, duration of infertility. However, patients with HP had a higher proportion of previous ectopic pregnancies (27.59 vs.9.48%, *P* = 0.010; OR 3.636, 95% CI 1.360–10.126) and multiple abortion history (≧2 times) (62.07 vs. 36.21%, *P* = 0.011; OR 2.883, 95% CI 1.244–6.680) than IUP. Furthermore, patients with HP had a higher proportion treated with ART due to tubal factor infertility (68.97 vs. 43.97%, *P* = 0.016; OR 2.832, 95% CI 1.189–6.746). Compared with patients in IUP group, those in HP group had a significantly higher spontaneous abortion rate (31.03 vs. 13.79%, *P* = 0.028; OR 2.832, 95% CI 1.189–6.746) and preterm delivery rate (25.00 vs. 7.00%, *P* = 0.035; OR 3.571, 95% CI 1.029–12.391). Patients with HP had a significantly lower live birth rate than those with IUP (58.62 vs. 78.45%, *P* = 0.028; OR 0.389, 95% CI 0.164–0.921). No major infant malformations were diagnosed (Table 1).

**Table 1:** Maternal characteristics and pregnancy outcome of study population.

	**HP (*N* = 29)**	**IUP (*N* = 116)**	***P*-value**	**OR (95% CI)**
Age (y)	32.15 ± 3.21	32.48 ± 3.15	0.624	
Body mass index (BMI), kg/m^2^	21.43 ± 2.15	22.77 ± 3.13	0.215	
Infertility diagnosis, *N* (%)			0.802	
Primary infertility	12 (41.38)	51 (46.74)		0.900 (0.394–2.053)
Secondary infertility	17 (58.62)	65 (53.26)		1.112 (0.487–2.536)
Duration of infertility (y)	3.87 ± 2.74	4.29 ± 2.60	0.415	
Previous abortions (≧2), *N* (%)	18 (62.07)	42 (36.21)	0.011	2.883 (1.244–6.680)
Previous ectopic pregnancies, *N* (%)	8 (27.59)	11 (9.48)	0.010	3.636 (1.360–10.126)
**Infertility diagnosis**, ***N*** **(%)**				
Tubal factor	20 (68.97)	51 (43.97)	0.016	2.832 (1.189–6.746)
Endometriosis	3 (10.34)	15 (12.93)	0.706	0.777 (0.209–2.886)
Non-tubal female factors	4 (13.79)	39 (33.62)	0.037	0.316 (0.103–0.972)
Male factor	2 (6.90)	11 (9.48)	0.663	0.707 (0.148–3.381)
**Pregnancy outcome**, ***N*** **(%)**				
Spontaneous abortion	9 (31.03)	16 (13.79)	0.028	2.813 (1.091–7.253)
Preterm delivery	5 (25.00)	7 (7.00)	0.035	3.571 (1.029–12.391)
Live births	17 (58.62)	91 (78.45)	0.028	0.389 (0.164–0.921)

*Values are expressed as mean ± standard deviation or n (%); OR (95% CI), odds ratio (95% confidence interval)*.

### ART Procedure Characteristics

In the present study, the number of embryos transferred in patients with HP was significantly greater than those with IUP (2.31 ± 0.47 vs. 1.96 ± 0.42, *p* = 0.029). Similarly, the incidence of ovarian hyperstimulation syndrome (OHSS) (24.13 vs. 6.90%, *p* = 0.006; OR 4.295 95% CI 1.411–13.275) in the HP group was higher in the IUP group. However, in patients with HP, there were no difference between thawed embryo transfer cycles and fresh embryo transfer cycles, and similar results were obtained when cleavage embryo and blastocyst transfer cycles were analyzed. In addition, no significant differences were detected in the number of retrieved oocytes, endometrial thickness, distance of air bubble to fundus between patients with HP and IUP (Table 2).

**Table 2:** ART procedure characteristics of study population.

	**HP (*N* = 29)**	**IUP (*N* = 116)**	***P*-value**	**OR (95% CI)**
Type of transfer cycles			0.803	
Fresh embryo	16 (55.17)	61 (52.59)		1.110 (0.490–2.513)
Frozen-thawed embryo	13 (44.83)	55 (47.41)		0.901 (0.398–2.041)
Number of oocytes retrieved	13.76 ± 4.92	12.98 ± 3.28	0.243	
Number of embryos transferred	2.31 ± 0.47	1.96 ± 0.42	0.029	
Stage of embryo			0.919	
Cleavage stage	23 (79.31)	91 (78.45)		1.053 (0.387–2.867)
Blastocyst stage	6 (20.69)	25 (21.55)		0.950 (0.349–2.585)
Endometrial thickness (mm)	10.20 ± 2.56	11.05 ± 1.64	0.502	
Distance of air bubble to fundus (mm)	17.55 ± 1.80	15.95 ± 2.15	0.272	
Ovarian hyperstimulation syndrome	7 (24.13)	8 (6.90)	0.006	4.295 (1.411–13.275)
β-hCG level (mIU/mL) after embryo transfer				
Day 14	399.10 ± 225.03	376.14 ± 161.27	0.599	
Day 18	1837.28 ± 1091.85	1867.95 ± 529.16	0.715	

*Values are expressed as mean ± standard deviation or n (%); OR (95% CI), odds ratio (95% confidence interval)*.

### Risk Factors Associated With HP

To assess the effects of different risk factors on incidence rate of HP after embryo transfer, multivariate logistic regression was used (Table 3). History of multiple abortions (≧2 times) (OR 3.031, 95% CI 1.087–8.453; *P* = 0.034), tubal infertility (OR 3.844, 95% CI 1.268–11.656; *P* = 0.017), previous ectopic pregnancies (OR 2.303, 95% CI 0.625–8.490; *P* = 0.021) and number of embryos transferred (OR 0.300, 95% CI 0.092–0.983; *P* = 0.037) were associated with an elevated proportion of HP following IVF/ICSI treatment. The incidence rate of HP was comparable in patients with cleavage embryo and blastocyst transfer. Interestingly, for all women involved in this research, endometrial thickness on the day of embryo transfer showed no difference, lacking association with heterotopic pregnancies.

**Table 3:** Risk factors associated with HP.

**Risk factors**	**OR (95% CI)**	***P*-value**
Previous ectopic pregnancies (yes/no)	2.303 (0.625–8.490)	0.021
Tubal factor (yes/no)	3.844 (1.268–11.656)	0.017
Previous abortion (≧2 times)	3.031(1.087–8.453)	0.034
Number of transferred embryos	0.300 (0.092–0.983)	0.037
Endometrial thickness	1.077 (0.832–1.394)	0.575
Stage of embryo (cleavage/blastocyst)	2.140 (0.551–8.308)	0.272

*OR (95% CI), odds ratio (95% confidence interval)*.

### Influence of Surgical Characteristics on Survival of Intrauterine Pregnancy Following Surgical Treatment of HP

**Figure 1:**
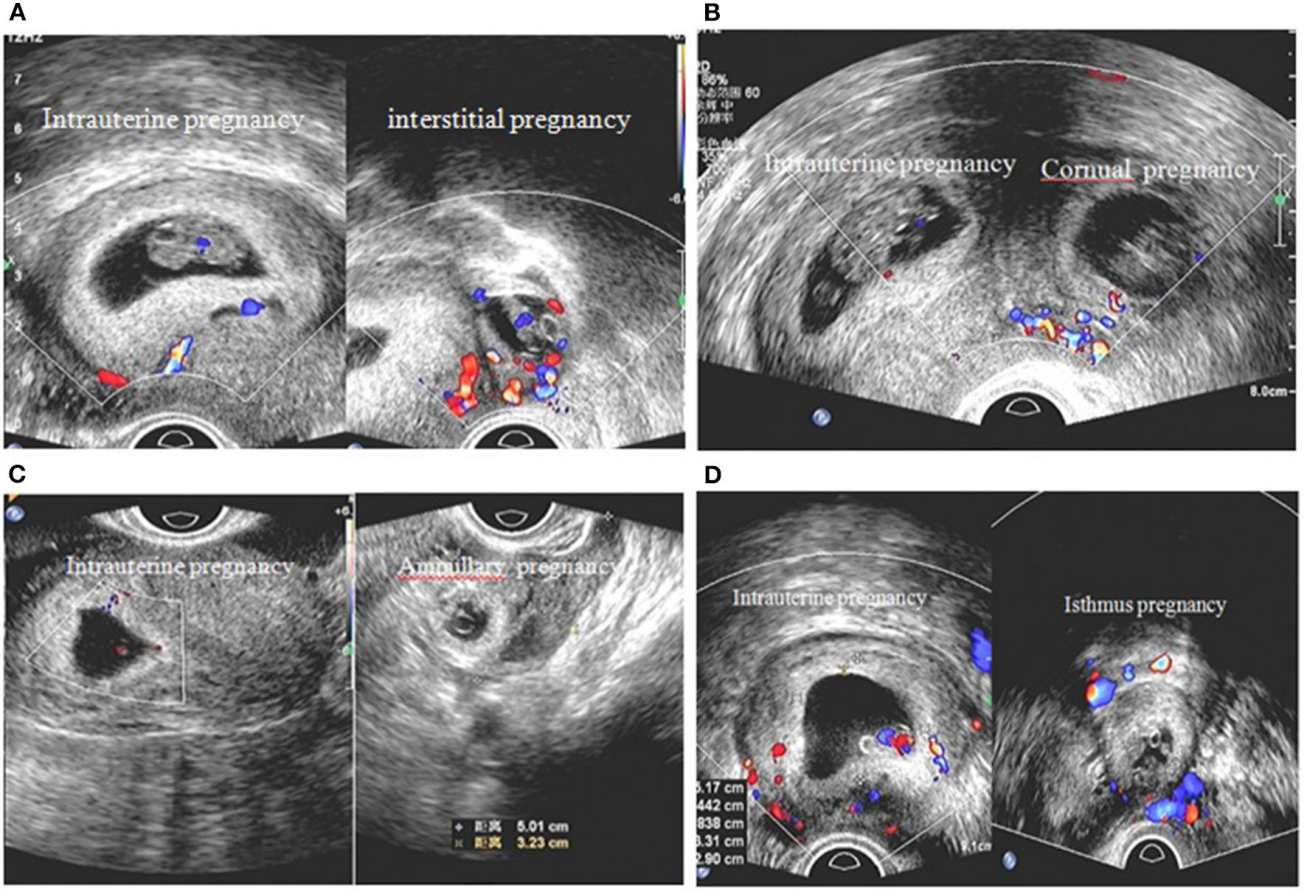
Ultrasound diagnosis of intrauterine pregnancy and location of ectopic pregnancy. Heterotopic pregnancy with different locations: **(A)** intrauterine pregnancy and left interstitial pregnancy **(B)** intrauterine pregnancy and right cornual pregnancy **(C)** intrauterine pregnancy and left fallopian ampullary pregnancy **(D)** intrauterine pregnancy and left fallopian isthmus pregnancy.

Twenty five patients with HP received surgical treatment on day of hospital admission. Among the patients, the ectopic gestation was in the ampulla in 18 (72%), the tubal interstitium in 3 (12%), and the tubal isthmus in 4 (16%) (Figure 1). The extrauterine gestation sac ruptured spontaneously in 3 patients. Among the 25 patients with HP, 24 (96%) underwent salpingectomy. Besides, the remaining one patient whose fallopian tubes had been removed was treated by placing a loop on stump of the tube. 20 (80%) underwent laparoscopic surgery, and 5 (20%) underwent laparotomy. No serious complications associated with surgery or anesthesia were reported.

To explore influence of surgical characteristics on survival of intrauterine pregnancy following surgical treatment of HP, an additional sub-analysis was restricted to patients treated with surgery. Table 4 shows the surviving rate of the intrauterine pregnancy according to different surgical characters. As shown in the table, while the duration of surgery increased, the surviving rate of the intrauterine pregnancy decreased significantly (100 vs. 75 vs. 33.33%, *p* = 0.034). The similar result was given when the effect of adnexal masses size on the intrauterine pregnancy was assessed. Furthermore, when the fallopian tube ruptured, the surviving rate of intrauterine pregnancy decreased to 33.33% (*P* = 0.013). When compared with other locations of ectopic pregnancy, the ampulla was strongly associated with a higher surviving rate of the intrauterine pregnancy (94.44%, *P* = 0.013) (Table 4).

**Table 4:** Influence of surgical characteristics on survival of intrauterine pregnancy following surgical treatment of HP.

	**Number of surgical treatment (*N* = 25)**	**Survival rate of intrauterine pregnancy (*N* = 20)**	***P*-value**
Gestational age at the time of surgery (day, calculated from the date of embryo transfer)			0.245
≦30	5	4 (80.00)	
31–56	16	14 (87.50)	
>56	4	2 (50.00)	
Position of ectopic pregnancy			0.013
Ampullar	18	17 (94.44)	
Interstitial	3	1 (66.67)	
Isthmus	4	2 (50.00)	
Method of surgery			0.252
Laparoscopy	20	17 (85.00)	
Laparotomy	5	3 (60.00)	
Duration of surgery(min)			0.034
≦30	10	10 (100.00)	
30–60	12	9 (75.00)	
>60	3	1 (33.33)	
sizes of the ectopic pregnancy lesions (cm)			0.013
≦5	18	17 (94.44)	
>5	4	2 (50.00)	
tubal rupture	3	1 (33.33)	

*Values are expressed as n (%)*.

## Discussion

Heterotopic pregnancies are extremely rare forms of ectopic pregnancy in obstetrics. However, in recent years the incidence rate has increased to 1% with widespread use of ART and HP is a life-threatening condition which have a maternal mortality rate eight times greater than tubal ectopic pregnancies (6). For this reason, early recognition and treatment is critical to improving the prognosis. Clinical features associated with HP vary widely among individuals. Some patients were asymptomatic, and others may experience acute abdominal pain, pelvic hemorrhage and hypovolemic shock. Though ectopic pregnancy and intrauterine pregnancy at the same time contribute to the serum β-hCG levels in patients with HP, the present study suggested no significant difference between the HP and IUP with aspect to blood level of β-hCG. β-hCG is usually low in ectopic pregnancies. However, it's probably unhelpful in diagnosis of heterotopic pregnancy, for they might indicate normal ranges in patients with HP. Transvaginal ultrasound provides a very important tool for the diagnosis of HP. However, the simultaneously presented concurrent intrauterine sac and bilaterally hyperstimulated ovaries greatly increased the difficulty in diagnosis. Given the above, diagnosis of HP is a major predicament for clinicians.

Many studies have shed light on the risk factors of HP over the past few years, with the hope of a clinical prediction model for it (4). In our study, history of multiple abortions, tubal infertility, previous ectopic pregnancies and multiple embryo-transfer were associated with an increased incidence of HP following IVF/ICSI treatment. Thus, heterotopic pregnancies should always be involved in differential diagnosis in symptomatic patients with risk factors suggested above. Furthermore, outpatient follow-up should be performed more frequently in patients with multiple embryo transfer, even though an intrauterine gestational sac is visible on ultrasound. A history of salpingitis and gross tubal damage in patients acts as a potential pathological mechanism of ectopic pregnancies following IVF and in natural conception (7, 8). The results are consistent with the present study which has shown that patients with history of tubal damage (salpingitis and previous ectopic pregnancies) are associated with increased incidence of ectopic pregnancy. A possible explanation is that altered tubal anatomy and function due to tubal damage may alter the mechanism of tubal transport and signaling molecules in tubal microenvironment which prevents embryonic implantation in the oviduct. Compared with other indications for IVF treatment, tubal indication is a major risk for HP in the current study. The same results were also presented in Li et al. study (9). The probable explanation of tubal pregnancies in those patients was that the embryos migrated into the oviduct after uterine placement, but the damaged tubal failed to transport the embryos back into the uterine.

The distance from the tip of transfer catheter to uterine fundus has also been evaluated as a potential risk factor for development of HP (10). In our center, distance of air bubble to fundus was controlled in 15–20 millimeters range, and the data suggested no significant difference between HP and IUP (17.55 ± 1.80 vs. 17.32 ± 1.75 mm, *p* = 0.55). The result was consistent with study by Fiçicioglu et al. (11) which reported that air bubble position too close to the fundus (10 mm) probably ended in a tubal pregnancy while at a distance of 15–20 millimeters would achieve a higher rate of embryo implantation. That is a possible explanation for the comparable data on distance of air bubble to fundus between the two groups in our center. In the present study, Logistic regression analysis on the number of embryos transferred and the risk of HP demonstrated that elective single embryo transfer may obtain 70% protection, reducing the incidence of HP (OR 0.300, 95% CI 0.092–0.983; *P* = 0.037). This finding confirms and expands that of a previous study which believed that the diagnosis of HP must be considered in patients with two or more embryos transferred in a cycle (12). Therefore, selective single-embryo transfer probably is a preferred choice for patients ≦40 years old for a decreased risk of HP. Overall, in the present study, we found some factors from the cause of infertility and specific characteristics of IVF and embryo-transfer techniques which increased the risk of HP following embryo transfer. Patients treated with IVF-ET should have more frequent ultrasound examinations early in pregnancy, for delay in diagnosis of HP is more common compared with ectopic pregnancy. Therefore, it's important to increase clinicians' awareness of HP though an intrauterine pregnancy is present, especially in patients with risk factors given above.

Surgery is still the most frequently chosen method of treatment with HP. The surgical approach is laparotomy and laparoscopy. The efficacy and safety of laparoscopic surgery during pregnancy is well certificated. It shows superiority over laparotomy approaches in postoperative recovery and subsequent reproductive outcomes (13). In the present study, 20 (80%) patients were treated by laparoscopy. The surgical procedures were slightly modified, avoiding both excessive manipulation and cannulation due to co-exsistance of the gestational sac in uterine. Among the 25 patients, 24 (96%) underwent salpingectomy. A possible explanation is that, compared with salpingostomy, salpingectomy gives the competitive advantages of shortening the operation time, decreasing rates of persistent trophoblast, reducing intraoperative stimulation of uterine and avoiding the possibility of another ectopic pregnancy (14). For the patients urgently needing a child, salpingectomy was acceptable if compensated by a higher survival rate of the intrauterine pregnancy following surgical treatment. The data suggested that patients with HP was more likely to end in miscarriage of the viable uterine pregnancy than those with IUP (31.03 vs. 13.79%, *P* = 0.028). In addition, HP was less likely to result in a live birth than IUP (58.62 vs. 78.45%, *P* = 0.028), probably due to the increased risk for miscarriages. It is important to explore this finding further, and the influence of the surgery on the pregnancy outcomes should be noted. Therefore, we analyzed survival factors of intrauterine pregnancy following surgical treatment of HP. We found that shorter operative duration, smaller size of the ectopic mass was associated with more chance to survive for the coexistent intrauterine pregnancy, and the prognosis would be better if ectopic pregnancies were in the ampulla of fallopian tube. As noted above, the factors are somewhat interrelated: a smaller size and ampulla located pregnancy involves a simpler surgical procedure and shorter duration of operation (salpingectomy), less manipulation of the uterus and surgical complications, which was a key point for a better reproductive outcome. This finding is consistent with previous research (15), suggesting that salpingectomy may be a preferred choice in women with HP following IVF-ET.

The study also has some limitations. Firstly, in addition to tubal factors, some other diseases of PID such as pelvic adhesions may also cause infertility. This research mainly studies the risk of HP and pregnancy outcomes; therefore, the above population is not included, which may lead to a confounding bias. Secondly, patients with HP are treated in the hospitals near the residence. The prognosis of HP is related to the surgical procedure; however, different surgeons may cause bias in the reproductive results.

In the current study, 3 patients had severe abdominal pain and massive pelvic hemorrhage due to tubal rupture. As a result, only one of them (33.3%) had the chance for an ongoing pregnancy after emergency surgery. Delayed diagnosis of HP in patients with a visible intrauterine pregnancy is life-threatening. It must be emphasized that for women treated with IVF-ET, even if an intrauterine gestational sac is confirmed, a high index of suspicion for HP is required, especially for these high-risk patients.

## Conclusion

HP is becoming more common with widespread use of ART. However, the most challenging aspect remains early diagnosis. Previous history of ectopic pregnancy, multiple abortions (≧2 times), and tubal indication are probable risk factors for HP in patients with IVF-ET treatment. Patients with these risk factors should be closely monitored by repeated ultrasonography after a positive pregnancy test. The surgical management of HP may result in a successful maternal outcome when early diagnosed. Finally, further expanded research is needed to explore how to decrease incidence rate of ectopic pregnancy after IVF-ET.

## Data Availability Statement

The original contributions presented in the study are included in the article/supplementary material, further inquiries can be directed to the corresponding author.

## Ethics Statement

The studies involving human participants were reviewed and approved by the Ethics Committee of Reproductive & Women-Children Hospital, Chengdu University of Traditional Chinese Medicine. The patients/participants provided their written informed consent to participate in this study.

## Author Contributions

SZ: programming the study, acquisition of data, drafting the article, and final approval of the version to be published. YF: the manuscript's conception and design and drafting the article. TD and LL: acquisition of data, drafting the article, and final approval of the version to be published. QZ: the manuscript's design and revising. All the authors contributed to the writing process of the manuscript and approved the final version.

## Funding

This study was supported by Key research and development projects of Science and Technology Department of Sichuan Provence (Grant No. 2022YFS0251), National Natural Science Foundation of China (Grant No. 82004176), and Xinglin Scholar Hospital Special Project of Chengdu University of Traditional Chinese Medicine (Grant No. 2020yky03).

## Conflict of Interest

The authors declare that the research was conducted in the absence of any commercial or financial relationships that could be construed as a potential conflict of interest.

## Publisher's Note

All claims expressed in this article are solely those of the authors and do not necessarily represent those of their affiliated organizations, or those of the publisher, the editors and the reviewers. Any product that may be evaluated in this article, or claim that may be made by its manufacturer, is not guaranteed or endorsed by the publisher.
